# Development of cancer surveillance guidelines in ataxia telangiectasia: A Delphi‐based consensus survey of international experts

**DOI:** 10.1002/cam4.6075

**Published:** 2023-06-02

**Authors:** Renata Neves, Blanca De Dios Perez, Rafal Panek, Sumit Jagani, Sophie Wilne, Jayesh M. Bhatt, Caterina Caputi, Emilia Cirillo, David J. Coman, Gregor Dückers, Donald L. Gilbert, Mary Kay Koenig, Lobna Mansour, Elizabeth McDermott, Micaela Pauni, Claudio Pignata, Susan L. Perlman, Oscar Porras, Mariela Betina Porto, Katherine Schon, Pere Soler‐Palacin, Sam Nick Russo, Masatoshi Takagi, Marc Tischkowitz, Claire Wainwright, Madhumita Dandapani, Cristine Glazebrook, Mohnish Suri, William P. Whitehouse, Robert A. Dineen

**Affiliations:** ^1^ Radiological Sciences, School of Medicine University of Nottingham Nottingham UK; ^2^ Department of Radiology Nottingham University Hospitals NHS Trust Nottingham UK; ^3^ Centre for Rehabilitation and Ageing Research, School of Medicine University of Nottingham Nottingham UK; ^4^ Medical Physics & Clinical Engineering Nottingham University Hospitals NHS Trust Nottingham UK; ^5^ Department of Paediatric Oncology Nottingham University Hospitals NHS Trust Nottingham UK; ^6^ Nottingham Children's Hospital, Nottingham University Hospitals NHS Trust Nottingham UK; ^7^ UK National Paediatric Ataxia Telangiectasia Clinic Nottingham University Hospitals NHS Trust Nottingham UK; ^8^ Department of Human Neuroscience Sapienza University Rome Italy; ^9^ Department of Translational Medical Sciences Federico II University Naples Italy; ^10^ Metabolic Medicine Queensland Children's Hospital Brisbane Queensland Australia; ^11^ School of Medicine University of Queensland, Brisbane Queensland Australia; ^12^ Childrens Hospital Helios Klinikum Krefeld Krefeld Germany; ^13^ Division of Neurology Cincinnati Children's Hospital Medical Center Cincinnati Ohio USA; ^14^ Department of Pediatrics University of Cincinnati College of Medicine Cincinnati Ohio USA; ^15^ Division of Child & Adolescent Neurology, Department of Pediatrics The University of Texas McGovern Medical School Houston Texas USA; ^16^ Center for the Treatment of Pediatric Neurodegenerative Disease The University of Texas McGovern Medical School Houston Texas USA; ^17^ Department of Pediatrics, Neuropediatric Unit Cairo University Children Hospital Cairo Egypt; ^18^ Clinical Immunology and Allergy Department Nottingham University Hospital NHS Trust Nottingham UK; ^19^ Neurologia Infantil Hospital Italiano de Buenos Aires Argentina; ^20^ Department of Neurology University of California Los Angeles California USA; ^21^ Pediatric Immunology and Rheumatology Department National Children's Hospital “Dr. Carlos Sáenz Herrera” San José Costa Rica; ^22^ Hospital Provincial de Rosario Santa Fe Argentina; ^23^ East Anglian Medical Genetics Service Cambridge University Hospitals NHS Foundation Trust Cambridge UK; ^24^ Pediatric Infectious Diseases and Immunodeficiencies Unit Hospital Universitari Vall d'Hebron Barcelona Spain; ^25^ Jeffrey Modell Diagnostic and Research Center for Primary Immunodeficiencies Barcelona Spain; ^26^ Universitat Autonoma de Barcelona Barcelona Spain; ^27^ Department of Pediatrics and Developmental Biology Tokyo Medical and Dental University Tokyo Japan; ^28^ Department of Medical Genetics, National Institute for Health Research Cambridge Biomedical Research Centre University of Cambridge Cambridge UK; ^29^ Department of Respiratory and Sleep Medicine Queensland Children's Hospital Brisbane Australia; ^30^ Children's Brain Tumour Research Centre University of Nottingham Nottingham UK; ^31^ Institute of Mental Health University of Nottingham Nottingham UK; ^32^ Nottingham Clinical Genetics Service Nottingham University Hospitals NHS Trust Nottingham UK; ^33^ Child Health, School of Medicine University of Nottingham Nottingham UK; ^34^ NIHR Nottingham Biomedical Research Centre Nottingham UK; ^35^ Sir Peter Mansfield Imaging Centre University of Nottingham Nottingham UK

**Keywords:** ataxia telangiectasia, cancer predisposition, cancer surveillance, guidelines, international survey, life‐limiting disease

## Abstract

**Background/Objectives:**

Ataxia telangiectasia (A‐T) is a multiorgan disorder with increased vulnerability to cancer. Despite this increased cancer risk, there are no widely accepted guidelines for cancer surveillance in people affected by A‐T. We aimed to understand the current international practice regarding cancer surveillance in A‐T and agreed‐upon approaches to develop cancer surveillance in A‐T.

**Design/Methods:**

We used a consensus development method, the e‐Delphi technique, comprising three rounds. Round 1 consisted of a Delphi questionnaire and a survey that collected the details of respondents' professional background, experience, and current practice of cancer surveillance in A‐T. Rounds 2 and 3 were designed based on previous rounds and modified according to the comments made by the panellists. The pre‐specified consensus threshold was ≥75% agreement.

**Results:**

Thirty‐five expert panellists from 13 countries completed the study. The survey indicated that the current practice of cancer surveillance varies widely between experts and centres'. Consensus was reached that evidence‐based guidelines are needed for cancer surveillance in people with A‐T, with separate recommendations for adults and children. Statements relating to the tests that should be included, the age for starting and stopping cancer surveillance and the optimal surveillance interval were also agreed upon, although in some areas, the consensus was that further research is needed.

**Conclusion:**

The international expert consensus statement confirms the need for evidence‐based cancer surveillance guidelines in A‐T, highlights key features that the guidelines should include, and identifies areas of uncertainty in the expert community. This elucidates current knowledge gaps and will inform the design of future clinical trials.

## Graphical abstract

Ataxia Telangiectasia (A‐T) is a multiorgan disorder with increased vulnerability to cancer. Despite this increased cancer risk, there are no widely accepted guidelines for cancer surveillance in people affected by A‐T. The international expert consensus statement developed in this study, confirms the need for evidence‐based cancer surveillance guidelines in A‐T, highlights key features that the guidelines should include, and identifies areas of uncertainty in the expert community.

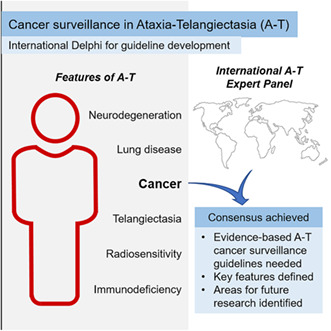

## 1 INTRODUCTION

Ataxia telangiectasia (A‐T) is an autosomal recessive disorder characterised by cerebellar degeneration, immunodeficiency, respiratory disease, dilated small blood vessels, radiosensitivity, and cancer susceptibility.^1^, ^2^, ^3^ A‐T is a complex disorder caused by mutations in the *ATM* (ataxia telangiectasia mutated) gene, which results in absent, non‐functioning or hypofunctioning *ATM* protein.^1^, ^2^, ^3^, ^4^ The *ATM* protein has roles in double‐stranded DNA repair and thus *ATM* variants lead to genomic instability with increased sensitivity to ionising radiation and elevated cancer risk (22%–24% cumulative incidence up to age 20 years).^1^, ^2^, ^3^, ^4^ People with classical A‐T have a more severe clinical phenotype due to either the complete absence of *ATM* protein or the production of mutant *ATM* protein with no kinase activity.^1^, ^5^, ^6^ People with variant A‐T have some residual *ATM* function and consequently a milder clinical phenotype with later age of onset, a slower rate of disease progression and a lower risk of developing cancer in childhood.^1^, ^5^, ^6^, ^7^ Classical A‐T manifests in early childhood (usually by the age of 2 years) with a life expectancy of around 25 years.^5^, ^6^, ^8^ Cancer and lung disease are the two leading causes of death.^8^, ^9^ Cancer in A‐T has been reported as early as 2 years and the median age of diagnosis of 12.5 years.^7^, ^10^

The Paediatric Cancer Working Group of the American Association for Cancer Research (AACR) recommends surveillance in cancer predisposition syndromes (CPS) with cancer risk above 5% (up to age 20 years),^11^ but specific evidence‐based guidance on cancer surveillance for A‐T is lacking.^9^, ^12^ The AACR Childhood Cancer Predisposition workshop report states that ‘Evidence‐based standards for cancer screening do not exist for patients with A‐T, particularly in childhood’ and recommends consideration of ‘Annual physical exam, complete blood count, and complete metabolic profile including lactate dehydrogenase’^4^ ^(*p2*)^. Van et al. (2017)^13^ provide broad guidance that ‘patients should be screened for malignancies periodically’ and that ‘Annual laboratory testing should at least include blood count and smear, immunoglobulin levels, M‐protein, and measurement of lactate dehydrogenase’, but the evidence base for this guidance is unclear. The guidance recommends that annual imaging surveillance be performed in adults (annual abdominal ultrasound and breast MRI over the age of 25 years) but does not include recommendations for imaging in children.^13^

Recently, the guidelines for cancer surveillance in some CPS, for example in Li‐Fraumeni syndrome (LFS), have changed due to new evidence‐based research.^7^, ^14^, ^15^, ^16^, ^17^, ^18^, ^19^ These guidelines include the recommendation for whole‐body imaging optimised for cancer detection, which is increasingly being used clinically for diagnosing and monitoring cancers and non‐cancer lesions.^20^, ^21^, ^22^, ^23^, ^24^, ^25^, ^26^, ^27^, ^28^, ^29^, ^30^ Modern magnetic resonance imaging (MRI) systems allow whole‐body imaging with relatively short acquisition times and provide excellent soft‐tissue contrast for lesion detection.^24^, ^27^, ^31^ Most importantly, MRI surveillance would not expose people with A‐T to the risks of ionising radiation.^24^, ^27^, ^31^

It has been shown recently that MRI can have an important role in the assessment of the respiratory tract in paediatric A‐T patients, which is commonly affected by a large spectrum of respiratory disorders, as well as the abdominal cavity.^32^

As a first step to designing a prospective clinical trial of cancer surveillance in people with A‐T, which will in turn lead to evidence‐based guidelines for cancer surveillance, we have undertaken an international survey of current practice and an e‐Delphi consensus‐finding exercise of clinical A‐T experts. The Delphi technique has been used in health research as a method that aims to achieve consensus on an important subject and to develop new concepts, especially when there is a lack of scientific evidence.^33^, ^34^, ^35^, ^36^ The theory behind this method assumes that the opinion generated in a group discussion is more valid than an individual opinion.^33^, ^36^ This work aims to find expert consensus regarding the need for evidence‐based guidelines for cancer surveillance in people with A‐T, to define the key features that should be included in the guidelines, and to identify areas of uncertainty that should be targeted by future research.

## 2 METHODS

### 2.1 The expert panel

We aimed to recruit a minimum of 15 experts for our e‐Delphi study, as previous research suggests that a sample of 12 experts can provide representative information.^34^, ^35^ Delphi panellists were recruited through the A‐T Clinical Research Network. An invitation email with the study description was sent to all members of this network. The first round questionnaire was sent to the members who expressed an interest in the study. All panellists were required to have 3 years post‐qualification experience in caring for people with A‐T and be currently employed in a clinical area related to A‐T.

### 2.2 Study design

The e‐Delphi study was conducted using the Joint Information Systems Committee (JISC) online surveys between October 2021 and April 2022. The number of e‐Delphi rounds was not pre‐specified. Panellists had 4 weeks to complete each survey round. Panellists were excluded from the next round if they did not complete the previous round. Panellists were informed that by completing each round, they were giving their consent to participate in the study. Information about panellists' specialisation and experience, and their current practice and guidelines regarding cancer surveillance in people with A‐T, was collected during the first e‐Delphi round.

In each e‐Delphi round, panellists were presented with either (1) a statement for which they were asked to indicate their level of agreement using a 7‐point Likert scale, (2) an agree/disagree question with an option to abstain or (3) a multiple choice question (MCQ) for which they were asked to indicate their preferred answer. Each statement or MCQ had an option to select either ‘do not feel able to answer’ or ‘other’ as an alternative response with a free‐text response box, allowing opinions to be provided that could be explored in subsequent rounds of the e‐Delphi. The statements and MCQs for the first round were developed by the research team following a literature review and were focused on the areas where the research team felt scientific evidence was lacking. Responses were analysed by three of the team members RN, BDP and RAD. The pre‐specified level of agreement for acceptance of a proposed statement was 75% of panellists, which is consistent with previous literature recommending this threshold.^33^, ^34^

Statements reaching the pre‐specified level of agreement were adopted into the final consensus statement unless panellists made an argument for improvement or clarification, in which case a revised version of the statement was put out to the panel in the next round.

Statements not reaching the pre‐specified level of agreement were revised by the study team according to the relevant free‐text responses and sent out for panel responses in the next round. The responses to the MCQs were used by the study team to propose new statements or revised MCQs with refinements made to the response options for review in the next round. The results of the previous round were provided to panellists where relevant.

## 3 RESULTS

### 3.1 Delphi panellists

**FIGURE 1:**
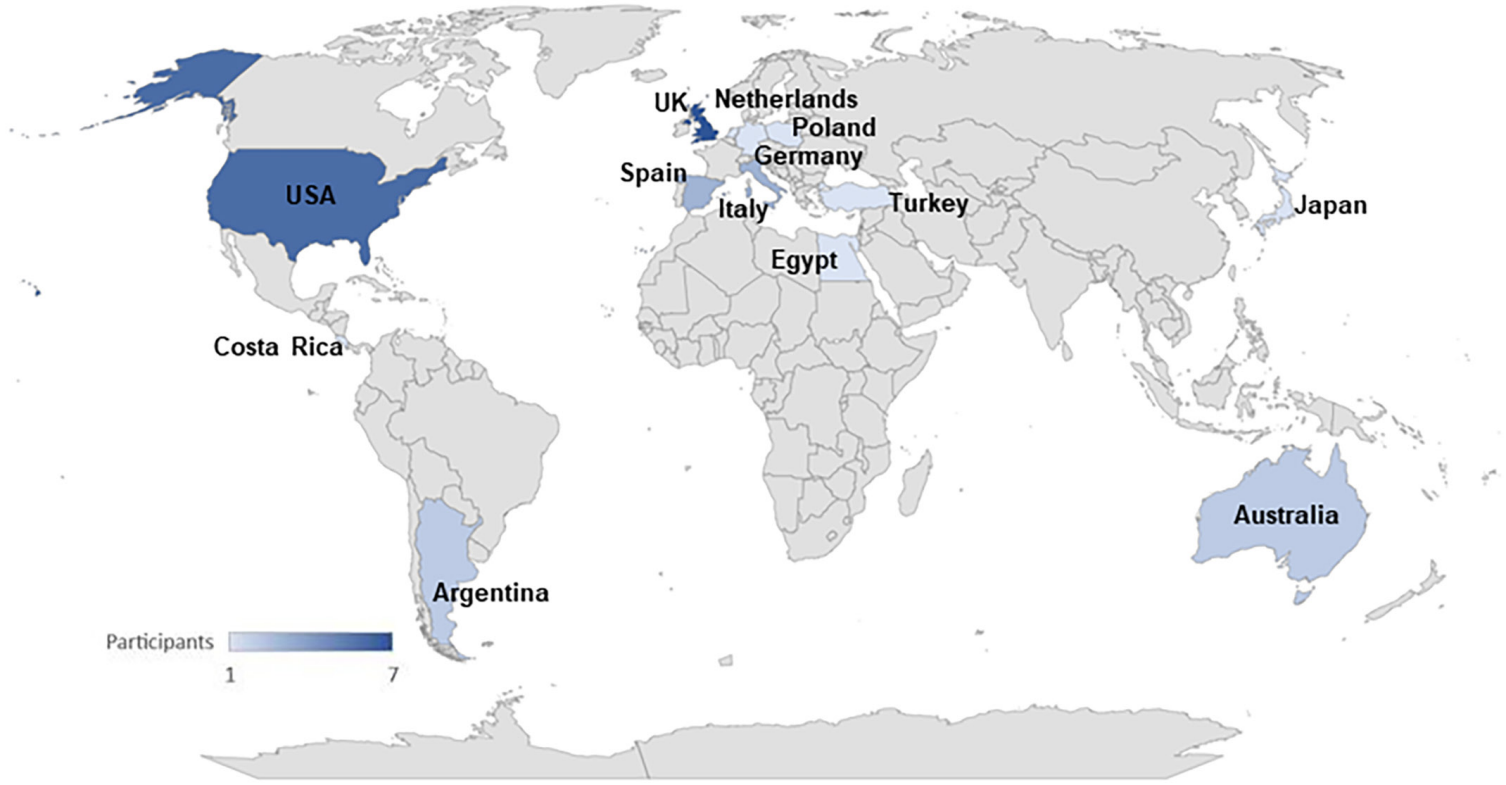
Geographic distribution of countries represented by the panellists.

Thirty‐five panellists from 13 countries agreed to participate (Figure 1). All panellists who completed the e‐Delphi Round 1 (30 panellists) provided details of professional background, experience and current practice relating to cancer surveillance in A‐T.

The majority of the panellists, who completed the survey and Round 1 of the e‐Delphi, were medical doctors (neurology, clinical genetics, haematology, oncology, immunology, paediatrics, respiratory medicine) and had worked with people with A‐T for ≥10 years (Table 1). Sixteen panellists (53%) were from a specialist clinic that provides care to people with A‐T and other similar disorders. Twenty‐four panellists (80%) reported caring for people with A‐T from the whole country where they are based.

**TABLE 1:** Panellists' professional experience.

Characteristics	e‐Delphi round 1	e‐Delphi round 2	e‐Delphi round 3
	(*n* = 30)	(*n* = 25)	(*n* = 24)
Professional background			
Medical	28 (93%)	23 (92%)	22 (92%)
Clinical scientist	2 (7%)	2 (8%)	2 (8%)
Years of working with people with A‐T			
3–4 years	4 (13%)	4 (16%)	4 (17%)
5–9 years	5 (17%)	4 (16%)	4 (17%)
10 or more years	21 (70%)	17 (68%)	16 (67%)
Age group of people with A‐T they work with			
Children	11 (37%)	8 (32%)	7 (29%)
Adult	2 (7%)	2 (8%)	2 (8%)
Both children and adult	17 (57%)	15 (60%)	15 (63%)

*Note*: Percentages shown are calculated relative to the number of experts participating in each round.

Fifteen of the 30 panellists who completed Round 1 reported conducting surveillance testing for cancer in people with A‐T (Table 2). Twenty‐seven (90%) and twenty‐four (80%) panellists mentioned that they do not have institutional and national guidelines regarding cancer surveillance in A‐T, respectively. When asked if they were aware of any guidelines, 12 panellists (40%) mentioned the UK A‐T Children Specialist Centre guidance^8^ and Li‐Fraumeni cancer surveillance guidelines.^14^, ^15^, ^16^, ^37^ Twenty‐eight panellists (93%) reported that implementing evidence‐based guidelines for cancer surveillance in A‐T would help with the management of people with A‐T.

**TABLE 2:** Current surveillance strategies reported by the panellists (*n* = 15).

Surveillance strategy	
Complete blood count and tumour markers (6–12 months)	67% (*n* = 10)
Breast screening—women with A‐T	13% (*n* = 2)
Physical examination (4–6 months)	33% (*n* = 5)
Patient and parent education regards signs and symptoms of cancer	7% (*n* = 1)
Surveillance questionnaire when in clinic	7% (*n* = 1)
Abdominal ultrasound—MRI if abnormalities are detected in ultrasound	27% (*n* = 4)
Oncological consultation	13% (*n* = 2)

**FIGURE 2:**
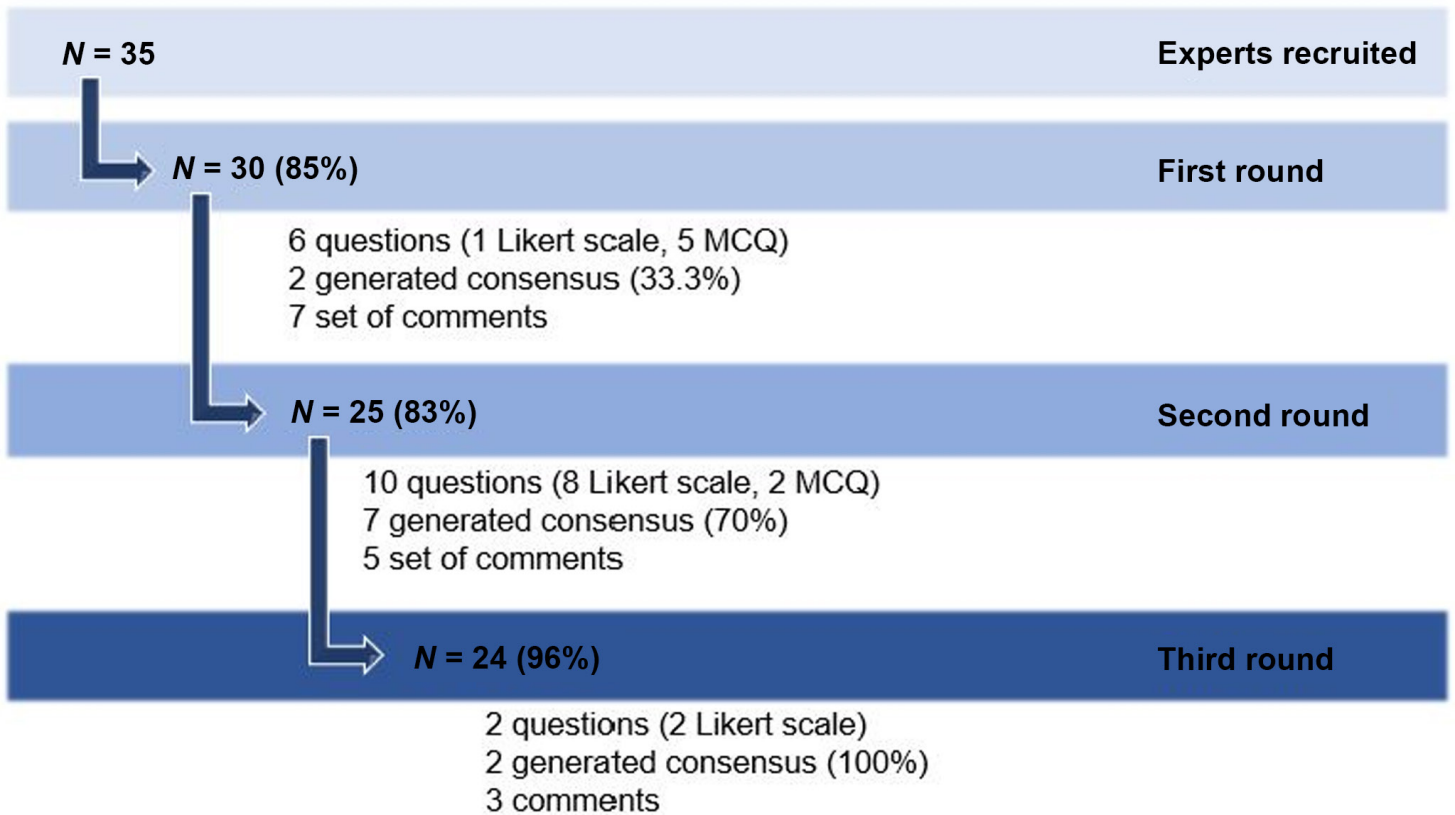
Flow diagram of the steps of the Delphi study: *N*, number of experts; MCQ, multiple choice question.

Three e‐Delphi rounds were required for the development of the final consensus statement. We obtained 30/35 response for Round 1 (85%), 25/30 for Round 2 (83%) and 24/25 for Round 3 (96%) (Figure 2).

Six questions were included in Round 1 (Data S1): one statement was accepted without change, one statement was resubmitted with minor language changes (based on panellists' comments) and four questions were modified and resubmitted in Round 2. These six questions were focused on topics in which the research team felt evidence was either absent or limited and were analysed as five main topics.

Ten statements were included in Round 2: seven were accepted unchanged and three were revised and resent in Round 3. Two statements were included in Round 3.

The consensus level improved from Round 1 to Round 3. In Round 1, consensus was reached in 2 (33%) out of 6 questions. In Round 2, consensus was reached in 7 (70%) out of 10 questions. In Round 3, consensus was reached in the 2 questions (100%) sent.

### 3.2 Areas of consensus and disagreement

#### 3.2.1 The need for guidelines for cancer surveillance in A‐T

In Round 1, the statement ‘Evidence‐based guidelines for cancer surveillance in people with A‐T are required’ received strong approval (90% strongly agree/agree). However, the free‐text comments indicated that some panellists felt the word ‘required’ at the end referred to the cancer surveillance tests per se, and hence that testing was mandated, rather than a guideline. In Round 2, this statement was refined to ‘Evidence‐based guidelines are needed for cancer surveillance in people with A‐T’ and received complete approval (100% strongly agree / agree).

#### 3.2.2 The need for specific guidelines based on age or type of A‐T

In round 1, the statement ‘Do you think that screening intervals would need to be different between children and adults with A‐T?’ did not achieve consensus (50% responded ‘Yes’, 20% responded ‘No’ and 30% responded ‘not able to answer this question’). The panellists who agreed with the statement mentioned that the types of tumours detected in A‐T can vary with age as well as the type of A‐T (classical or variant). The panellists who selected ‘No’ argued that cancer surveillance guidelines in A‐T would be useful for both age groups because the risk of developing cancer exists in both populations. Two statements were made based on these comments and were sent in the next round. In Round 2, the statement ‘Within these guidelines, separate recommendations should be developed for adults and children with A‐T’ received strong approval (88% agree). However, the statement ‘Within these guidelines, separate recommendations should be developed for people with classical and variant A‐T’ did not reach consensus (40% agree, 16% disagree, and 44% neither agree nor disagree). The panellists commented that there is no available evidence to support or not support this statement. Therefore, a new statement was developed ‘Further research is needed to understand whether separate guidelines for people with classical and variant A‐T are needed’ and sent in Round 3, which received complete approval (100% agree).

#### 3.2.3 The tests for inclusion in the guidelines

In Round 1, the statement related to the non‐imaging tests that should be included in cancer surveillance guidelines did not reach full consensus. There was strong approval (90%) for the inclusion of complete blood count (CBC) and blood film. However, the other suggested blood tests (liver function tests, αFP, βHCG, LDH and Epstein–Barr virus serology) did not achieve consensus. Therefore, three statements were developed and sent in Round 2. The consensus was reached in these three statements: ‘Complete (full) blood count should be included in A‐T cancer surveillance guidelines, with blood film (smear) performed if abnormal white cell counts or cytopenias are detected’ (96% agree); ‘Further research is needed to allow the optimal selection of blood tests for inclusion in guidelines for cancer surveillance for people with A‐T’ (84% agree); ‘Both imaging and non‐imaging tests (such as blood tests) for cancer surveillance are likely to be included in the guidelines, but the recommendations need to be flexible to allow different diagnostic tests based on local availability’ (92% agree).

#### 3.2.4 The age for starting and stopping cancer surveillance

In Round 1, the question ‘Do you think there should be an age at which cancer surveillance stops?’ achieved the consensus level, with 86% of the panellists indicating that there should be no age at which cancer surveillance stops. However, there was no consensus regarding the age at which cancer surveillance should start. The panellists also commented that the age for commencing cancer surveillance might be different for imaging and blood tests. Based on this, two MCQs were developed and included in Round 2. However, these MCQs regarding ‘age of commencing blood tests for cancer surveillance in A‐T’ and ‘age of commencing imaging tests for cancer surveillance in A‐T’ did not achieve consensus. Therefore, a new statement was developed for Round 3: ‘Further research is needed to understand the optimal age for commencing imaging and blood tests for cancer surveillance in A‐T’, which received strong approval (92% agree).

#### 3.2.5 The optimal interval for performing cancer surveillance tests

In Round 1, the statements regarding the optimum interval for performing surveillance tests and whether it should be different between children and adults with A‐T did not achieve consensus. More than 75% of panellists selected the options of surveillance at least once every year, more precisely 55% selected an interval of 1 year and 21% selected an interval of 6 months. The comments made by the panellists suggested that blood tests could be performed more frequently than imaging tests and the frequency of surveillance testing may need to be different not only between adults and children but also for people with classic and variant A‐T. Therefore, two statements were developed ‘Surveillance testing for cancer in people with A‐T is likely to be required at least annually, but further research is needed to allow optimal selection of surveillance interval for children and adults, and for people with classical and variant A‐T’ and ‘The surveillance interval may vary depending on the test, with blood tests being performed more frequently than imaging tests, but further research is needed to establish the optimal interval for different types of cancer surveillance testing’, with both achieving consensus in Round 2 (88% and 96% agree, respectively).

### 3.3 The final consensus statement

Following the three e‐Delphi rounds, the final consensus statements are:

1. ‘Evidence‐based guidelines are needed for cancer surveillance in people with A‐T.’
2. ‘Within these guidelines, separate recommendations should be developed for adults and children with A‐T.’
3. ‘Further research is needed to understand whether separate guidelines for people with classic and variant A‐T are needed.’
4. ‘Both imaging and non‐imaging tests (such as blood tests) for cancer surveillance are likely to be included in the guidelines, but the recommendations need to be flexible to allow different diagnostic tests based on local availability.’
5. ‘Complete (full) blood count should be included in A‐T cancer surveillance guidelines, with blood film (smear) performed if abnormal white cell counts or cytopenias are detected. Further research is needed to allow the optimal selection of blood tests for inclusion in guidelines for cancer surveillance for people with A‐T.’
6. ‘Surveillance testing for cancer in people with A‐T is likely to be required at least annually, but further research is needed to allow optimal selection of surveillance interval for children and adults, and for people with classical and variant A‐T.’
7. ‘The surveillance interval may vary depending on the test, with blood tests being performed more frequently than imaging tests, but further research is needed to establish the optimal interval for different types of cancer surveillance testing.’
8. ‘Further research is needed to understand the optimal age for commencing imaging and blood tests for cancer surveillance in A‐T. There should be no set age at which cancer surveillance in A‐T stops.’

## 4 DISCUSSION

This study used the e‐Delphi method to form a consensus statement regarding cancer surveillance in A‐T globally. It should be highlighted that 35 panellists were recruited from six continents, which helped to understand the current practice for cancer surveillance in A‐T worldwide. Furthermore, the final statement produced not only provides some guidance about important points that should be considered for cancer surveillance in A‐T, but also identified knowledge gaps that need to be addressed by future research.

Most experts included in this study care for people with A‐T in the whole country in which they are based and more than half of the experts work in a specialist clinic that includes A‐T and other similar disorders, which shows the rarity of this disorder. Most panellists, including those who mentioned performing cancer surveillance tests, confirmed that there are no institutional or national guidelines for cancer detection in A‐T. Indeed, some panellists mentioned that the UK A‐T children Specialist Centre guidance discusses the increased cancer risk and recommendations on treatment of cancer, but does not include cancer surveillance guidelines as such. The only surveillance guidelines highlighted by some panellists were the breast screening guidelines for women with A‐T.^38^ The LFS cancer surveillance protocol was also mentioned but its guidelines are not specific to A‐T.

There was a strong immediate agreement regarding the need for cancer surveillance guidelines in A‐T and the absence of an age limit for such a surveillance protocol. These results suggested that clinicians recognise the importance of cancer surveillance and the lack of evidence to date about how to best conduct this surveillance. In fact, the lack of guidelines for cancer surveillance in A‐T has been reported by several authors.^4^, ^9^, ^12^ The panellists also agreed that these recommendations should be different for children and adults with A‐T. Although there is limited evidence to date, this consensus may have been driven by the fact that different types of cancer are more likely at different ages (Table 3).

**TABLE 3:** Malignancies occurring in A‐T.^7^, ^9^, ^10^, ^39^

A‐T subtype	Paediatric	Adult
Classical	Lymphoid Acute lymphoblastic leukaemia Non‐hodgkin lymphoma Hodgkin lymphoma Burkitt lymphoma Other rare lymphoma types Non‐lymphoid Hepatocellular carcinoma Brain (glioma, medulloblastoma) Others—dermatofibrosarcoma, renal tumours, gastrointestinal tumours	Lymphoid Prolymphocytic leukaemia Others (rare)—Non‐hodgkin lymphoma, acute lymphoblastic leukaemia Non‐lymphoid Breast cancer Thyroid cancer Others—pancreatic carcinoma, testicular seminoma, ovarian
Variant	Lymphoid (Rare) Non‐hodgkin lymphoma, acute lymphoblastic leukaemia	Lymphoid (Rare) Prolymphocytic leukaemia, acute lymphoblastic leukaemia, myeloma Non‐lymphoid Breast cancer Thyroid cancer Gastrointestinal tumours Renal tumours

Another point that showed a strong agreement was the need to include imaging and non‐imaging tests in the cancer surveillance guidelines in A‐T, which follows the cancer surveillance recommendations of other CPS similar to A‐T.^4^, ^18^, ^19^

This study demonstrated that due to the limited evidence,^4^ there were points where an agreement was not obtained among the panellists, which highlights the need of gathering more evidence in the A‐T field. These points related to the frequency of the surveillance tests (imaging and non‐imaging), the age of commencing surveillance in A‐T, the optimal selection of blood tests and whether the cancer surveillance guidelines should be tailored according to the type of A‐T. The panellists agreed that specific research projects need to be conducted to obtain more data that could guide the development of guidelines for cancer surveillance in A‐T.

One strength of this study is the representative expert panel, which includes a large number of global experts with more than 10 years of experience in caring for people with A‐T. It is also important to highlight that the response rate was above 80% in all rounds. A limitation that should be considered is that the responses given by the experts could have been influenced by how they interpreted the statements. However, the experts were allowed to provide feedback on all the statements, which were then analysed and, in some cases, incorporated into the statements given in subsequent rounds. Nevertheless, and in the interest of keeping the e‐Delphi process manageable, it was not always possible to fully explore all the different recommendations made by the panellists in subsequent e‐Delphi rounds.

In conclusion, we provide an international expert consensus statement that strongly supports the development of evidence‐based cancer surveillance guidelines in A‐T, highlighting key features that the guidelines should include and identifying areas where there is uncertainty in the expert community. This provides the basis for the design of prospective clinical trials of cancer surveillance in A‐T, and points researchers towards knowledge gaps in the implementation of cancer surveillance in A‐T, which should be targeted by future research.

## AUTHOR CONTRIBUTIONS

**William Whitehouse:** Conceptualization (equal); funding acquisition (equal); investigation (equal); writing – review and editing (equal). **Sumit Jagani:** Conceptualization (equal); funding acquisition (equal); writing – review and editing (equal). **Susan Perlman:** Investigation (equal); writing – review and editing (equal). **Sophie Wilne:** Conceptualization (equal); funding acquisition (equal); writing – review and editing (equal). **Sam Nick Russo:** Investigation (equal); writing – review and editing (equal). **Robert Dineen:** Conceptualization (equal); data curation (equal); formal analysis (equal); funding acquisition (equal); investigation (equal); methodology (equal); project administration (equal); software (equal); supervision (equal); validation (equal); visualization (equal); writing – review and editing (equal). **Renata Neves:** Data curation (equal); formal analysis (equal); investigation (equal); methodology (equal); project administration (equal); resources (equal); software (equal); validation (equal); visualization (equal); writing – original draft (lead); writing – review and editing (equal). **Rafal Panek:** Conceptualization (equal); funding acquisition (equal); writing – review and editing (equal). **Claudio Pignata:** Investigation (equal); writing – review and editing (equal). **Pere Soler Palacin:** Investigation (equal); writing – review and editing (equal). **Oscar Porras:** Investigation (equal); writing – review and editing (equal). **Mohnish Suri:** Conceptualization (equal); funding acquisition (equal); investigation (equal); writing – review and editing (equal). **Micaela Pauni:** Investigation (equal); writing – review and editing (equal). **Marc Tischkowitz:** Investigation (equal); writing – review and editing (equal). **Mary Kay Koenig:** Investigation (equal); writing – review and editing (equal). **Mariela Betina Porto:** Investigation (equal); writing – review and editing (equal). **Lobna AbdelGawad Mansour:** Investigation (equal); writing – review and editing (equal). **Madhumita Dandapani:** Conceptualization (equal); funding acquisition (equal); writing – review and editing (equal). **Masatoshi Takagi:** Investigation (equal); writing – review and editing (equal). **Katherine Schon:** Investigation (equal); writing – review and editing (equal). **Jayesh Bhatt:** Investigation (equal); writing – review and editing (equal). **Gregor Dückers:** Investigation (equal); writing – review and editing (equal). **Emilia Cirillo:** Funding acquisition (equal); writing – review and editing (equal). **Elizabeth McDermott:** Investigation (equal); writing – review and editing (equal). **Donald Gilbert:** Investigation (equal); writing – review and editing (equal). **David Coman:** Investigation (equal); writing – review and editing (equal). **Cristine Glazebrook:** Conceptualization (equal); funding acquisition (equal); writing – review and editing (equal). **Claire Wainwright:** Investigation (equal); writing – review and editing (equal). **Caterina Caputi:** Investigation (equal); writing – review and editing (equal). **Blanca De Dios Perez:** Data curation (equal); formal analysis (equal); investigation (equal); methodology (equal); project administration (equal); software (equal); validation (equal); visualization (equal); writing – review and editing (equal).

## FUNDING INFORMATION

This study is funded by a grant from Action for A‐T (ref. 20NOT05). Renata Neves is supported by a Doctoral Fellowship awarded by the College of Radiographers (ref. DF021). This study is supported by the National Institute for Health Research (NIHR) Applied Research Collaboration East Midlands (ARC EM). The views expressed are those of the author(s) and not necessarily those of the NIHR or the Department of Health and Social Care.

## CONFLICT OF INTEREST STATEMENT

The authors declare no conflict of interest.

## Supporting information


Data S1.

## Data Availability

The data that support the findings of this study are available from the corresponding author upon reasonable request.
