# Supplementary material for: Development of cancer surveillance guidelines in ataxia telangiectasia: A Delphi‐based consensus survey of international experts
Source: Cancer Med. 2023 Jun 2;12(13):14663–73. doi: 10.1002/cam4.6075 (PMC10358231; doi:10.1002/cam4.6075)
Supplement: Supplementary file 1 — Data S1. [file CAM4-12-14663-s001.docx]

**Development of cancer surveillance guidelines in Ataxia Telangiectasia: A Delphi-based consensus survey of international experts**

**Supplementary file 1**

The Delphi questionnaire will consist of 6 questions with different formats.

The results collected on this round will be provided in the next round.

*Question 1*

| Do you agree with the following statement: "Evidence-based guidelines for cancer  surveillance in people with A-T are required" **Required* | | | | | | | |
| --- | --- | --- | --- | --- | --- | --- | --- |
|  | Strongly disagree | Disagree | Somewhat disagree | Neither agree nor disagree | Somewhat agree | Agree | Strongly agree |
| Level of agreement |  |  |  |  |  |  |  |
| Are there any suggestions you have for amending this statement? *Optional* | | | | | | | |
|  | | | | | | | |

*Question 2*

| We are conducting a feasibility trial of whole body MRI for cancer detection in people with AT. If this is successful, we hope to conduct a prospective trial of cancer screening in A-T, which will contribute to the development of evidence-based guideline. What other tests do you think should be included in a cancer surveillance programme in A-T? **Required* | |
| --- | --- |
|  | Full blood count and blood film |
|  | Liver function tests |
|  | Alpha fetoprotein |
|  | Beta Human Chorionic Gonadotrophin |
|  | Lactate Dehydrogenase |
|  | Epstein-Barr Virus |
|  | I do not feel able to answer this question |
|  | Other |
| Please specify | |
|  | |

*Question 3*

| *Based on your experience of caring for people with A-T, what do you think the optimum interval for performing screening test would be? *Required* | |
| --- | --- |
|  | 6 months |
|  | 1 year |
|  | 2 years |
|  | 3 years |
|  | 5 years |
|  | I do not feel able to answer this question |
|  | Other – please specify |
|  | |

*Question 4*

| *Do you think that screening intervals would need to be different between children and adults*  *with A-T *Required* | |
| --- | --- |
|  | Yes |
| Please explain further | |
|  | |
|  | No |
| Please explain further | |
|  | |
|  | I do not feel able to answer this question |

*Question 5*

| *Based on your experience of caring for people with A-T, at what age do you think cancer*  *screening should commence? *Required* | |
| --- | --- |
|  | Birth |
|  | From 2 years |
|  | From 3 years |
|  | From 5 years |
|  | From 10 years |
|  | I do not feel able to answer this question |
|  | Other – please specify |
|  | |

*Question 6*

| *Do you think there should be an age at which cancer surveillance stops? *Required* | |
| --- | --- |
|  | Yes |
|  | No |
|  | I do not feel able to answer this question |
| Please state age and rationale | |
|  | |

**Thank you!**

Thank you for completing this first round. The data will now be analysed.

The next round will be sent out once the analysis is complete. The feedback from this round will be presented in the next round.
